# Systematic Review of the Link Between Social Cognition and Suicidal Ideation and Behavior in People With Serious Mental Illness

**DOI:** 10.1093/schizbullopen/sgae007

**Published:** 2024-03-27

**Authors:** Emma M Parrish, Lisa Steenkamp, Samantha A Chalker, Raeanne C Moore, Amy Pinkham, Colin A Depp

**Affiliations:** San Diego State University/University of California San Diego Joint Doctoral Program in Clinical Psychology, San Diego, CA, USA; Department of Psychiatry, University of California San Diego, San Diego, CA, USA; Department of Child and Adolescent Psychiatry/Psychology, Erasmus MC Sophia Children’s Hospital, Rotterdam, the Netherlands; Department of Psychiatry, University of California San Diego, San Diego, CA, USA; Veterans Affairs San Diego Healthcare System, San Diego, CA, USA; Department of Psychiatry, University of California San Diego, San Diego, CA, USA; Department of Psychology, The University of Texas at Dallas, Dallas, TX, USA; Department of Psychiatry, University of California San Diego, San Diego, CA, USA; Veterans Affairs San Diego Healthcare System, San Diego, CA, USA

**Keywords:** social cognition, theory of mind, attributional bias, emotion recognition, schizophrenia, bipolar disorder, serious mental illness, suicide

## Abstract

**Background and Hypothesis:**

People with serious mental illness (SMI; psychotic and affective disorders with psychosis) are at an increased risk of suicide, yet there is limited research on the correlates of suicide in SMI. Social cognitive impairments are common among people with SMI and several studies have examined social cognition and suicidal ideation (SI) and behavior. This systematic review aims to evaluate the links between various domains of social cognition, SI, and suicidal behavior in SMI.

**Study Design:**

Electronic databases (PubMed and PsycInfo) were searched through June 2023. Records obtained through this search (*N* = 618) were screened by 2 independent reviewers according to inclusion criteria. Relevant data were extracted, and study quality was assessed.

**Study Results:**

Studies (*N* = 16) from 12 independent samples were included in the systematic review (*N* = 2631, sample sizes ranged from *N* = 20 to *N* = 593). Assessments of social cognition and SI and behavior varied widely between studies. Broadly, effects were mixed. Better emotion recognition of negative affect was linked to SI and a history of suicide attempts, though there is little consistent evidence for the relationship of emotion recognition and SI or behavior. On the other hand, better theory of mind ability was linked to SI and a history of suicide attempts. Furthermore, negative attributional bias was linked to current SI, but not a history of SI or attempt.

**Conclusions:**

This review suggests mixed associations between social cognition, SI, and behavior in SMI. Future research should evaluate additional mediators and moderators of social cognition and suicide, employing prospective designs.

## Introduction

Compared to the general population and others with mental health conditions, people with serious mental illness (SMI; ie, psychotic and affective disorders with psychosis) are at increased risk of suicidal ideation (SI) and suicidal behavior (SB).^1–8^ Factors contributing to SI and SB may be unique among people with SMI, including the influence of command hallucinations^9^ and the use of different and potentially more lethal means in suicide attempts.^10^ Even with this increased risk and unique factors contributing to suicide, there is little understanding of the determinants of SI and SB in SMI.^11,12^ Indeed, people with psychosis are often excluded from suicide-focused intervention trials, and there are few developed suicide-focused interventions for people with SMI.^13,14^ Thus, there is a need to identify constructs that may relate to suicide in SMI that may serve as modifiable treatment targets for this at-risk population.

Social cognition, or how individuals perceive, understand, and apply social information,^15^ represents a potentially important determinant of suicide in psychosis. Social cognitive impairments are common in people with schizophrenia^15,16^ and bipolar disorder.^17^ These deficits in cognition are often related to paranoid or persecutory delusions,^18^ and are linked to functional outcomes including but not limited to social skills, social relationships, and community functioning.^15,16,19^ There are 4 major areas of social cognitive abilities that may be impaired among people with SMI: emotion processing, social perception, theory of mind, and attributional style.^15,20,21^ These constructs represent related, but independent domains that may be linked to suicide risk in SMI. First, emotion processing involves skills related to perceiving, recognizing, and processing emotions.^15^ These skills can be relatively basic, such as emotion recognition, or complex, such as emotion regulation.^15^ Second, social perception involves the ability to decipher others’ social cues while incorporating contextual information in order to understand individuals’ relationships and roles.^15,21–23^ Third, theory of mind is the ability to infer others’ inner mental states and deduce others’ thoughts and intentions.^15,24^ Theory of mind can include skills such as picking up hints and interpreting metaphors, and is also known as “mental state attribution,” “mentalizing,” or “cognitive empathy.”^15,25^ Fourth, attributional style or bias refers to how 1 interprets and attributes or misattributes intention to social interactions.^15^ For instance, common attributional styles in schizophrenia are increased negative perceptions of events or others,^16^ or attributing hostility to the neutral actions of another person.^26^ All 4 of these major social cognitive domains are impaired in SMI,^15,16,27,28^ and generally studied in SMI as they relate to social functioning.^15–17,19,23^

### Social Cognitive Impairments and Suicide

The link between emotion recognition, theory of mind, SI, and SB is established in broader clinical populations. Emotion recognition deficits have been linked to suicide attempts in older adults.^29^ Additionally, a meta-analysis found evidence for a relationship between theory of mind impairments and SI and SB in a broad clinical population.^30^ Another meta-analysis of neurocognitive impairments and SI or SB among individuals with a first episode of psychosis (FEP) found that deficits in theory of mind were linked to suicide attempts.^31^ Though neurocognition has been linked to SI and SB in the general population and in SMI, social cognition is an independent construct and largely unrelated to neurocognition.^32^ Though neurocognition has been linked to SI and SB in the general population and in SMI, social cognition is an independent construct and largely unrelated to neurocognition.^32^ Thus, social cognition may be a unique treatment target due to its potential connection to interpersonal risk factors related to suicide, such as perceived burdensomeness and thwarted belongingness.^33^

The evidence that these social cognitive processes may be linked to suicide in SMI is sparser than in other populations and the conclusions have been inconsistent. While meta-analyses have examined the link between theory of mind, SI, and SB in the general population^30^ and the link of broad neurocognition, SI, and SB in FEP,^31^ no studies have examined the evidence for a link between social cognition, SI, and SB in aggregate. There needs to be an understanding of the relationship of social cognition to suicide, particularly a nuanced understanding of how specific social cognitive domains relate to SI and SB among people with SMI. If there is a connection between social cognition and SI and SB, the improvement of social cognition could impact SI and SB in this population, either through existing interventions that target social cognition in SMI^34^ or through a novel suicide-prevention intervention.

We conducted a systematic review to determine whether and which social cognitive constructs were associated with past and current SI and SB in SMI. This systematic review investigates 2 main aims: (1) to understand the relationship of social cognition to SI in SMI, and (2) to understand the relationship of social cognition to SB in SMI.

## Methods

This systematic review was conducted in accordance with current Preferred Reporting Items for Systematic Reviews and Meta-Analyses (PRISMA) guidelines.^35^

### Search Strategy

PubMed and PsycInfo databases were searched for articles through April 14, 2022, as well as an updated search for articles published from 2022 to 2023 conducted on June 12, 2023 (see supplementary materials for search terms).

### Screening and Study Selection Criteria

All papers were screened by 2 different authors (E.P. and L.S.). Rayyan software was used to conduct a 3-phase study screening and selection process.^36^ In phase 1, authors screened titles and abstracts from the initial search for general eligibility. In phase 2, authors obtained full texts of articles included in phase 1 and evaluated articles using strict inclusion and exclusion criteria. Inclusion criteria were: (1) written in English; (2) all participants had a diagnosis of a SMI (ie, schizophrenia, schizoaffective disorder, bipolar disorder with psychotic features, major depressive disorder with psychotic features) according to Diagnostic and Statistical Manual 5 or International Classification of Diseases 10 criteria, excluding control groups, and including FEP samples; (3) included a measure of social cognition (ie, emotion perception, social perception, theory of mind, attributional bias, or any alternate terms used to describe these constructs); (4) included a measure of SI and/or SB; and (5) tested the relationship between a social cognition and a suicide variable. Papers were excluded from the review if they included topics adjacent to social cognition, but not a social cognitive task (eg, “social perception” referring to public attitudes about suicide, “social functioning”), or if they included participants who experienced psychotic-like symptoms rather than met full diagnostic criteria. Studies including participants at clinical high risk for psychosis were also excluded. Non-peer-reviewed studies were considered for inclusion in this study, but reports were excluded if they were non-empirical or conference abstracts.

Following the identification of included studies in phase 2, phase 3 of the screening process included manual reference searches of included articles and cited papers through Google Scholar, in a “backward” and “forward” snowballing method.^37^ Additionally, 2 relevant meta-analyses identified in the search were screened for relevant articles.^30,31^

### Data Items Collected

See table 1 for sample characteristics, design, objective, measurement, and key findings. Measures of SI and/or behavior and social cognition were extracted. If characteristics were reported separately (eg, separate mean ages reported for those with and without a suicide attempt), group characteristics were combined. Additionally, outcomes concerning case-control comparisons, functional characteristics of the brain, and neuroimaging findings were not collected. Next, information about the study design and objective were collected along with key findings of the study relevant to the aims of this review.

**Table 1.** Studies Reporting on Emotion Recognition/Processing: Sample Characteristics, Design, Objective, Measurement, and Key Findings

Study and Country of Origin	Sample Size and Characteristics of Group With Psychotic Disorder	Design	Quality Rating	Measured SI and/or Behavior	Measure of SI and/or Behavior	Measure of Emotion Recognition/Processing	Key Findings—Relationship Between SI and Behavior
Comparelli et al., 2017^38^ (Italy)^a^	*N* = 86; Mean Age = 36.3, *SD* = 10.8; 27.9% Female; “Established diagnosis of schizophrenia”	CS	6	SI	Columbia Suicide Severity Rating Scale (Interview-Rated)—Lifetime SI	MCCB Social Cognitive Measure—Mayer-Salovey Caruso Emotional Intelligence Test (MSCEIT)	MSCEIT impairment was correlated with greater SI severity (*r* = 0.329, *P* < .05) No significant group-level difference in MSCEIT performance between people with and without SI A stepwise regression showed that social cognition explained 44% of the variance in SI severity when controlling for positive symptoms (*B* = −0.470, *P* = .015, Adj. *R*^2^ = 0.441)
Cuesta et al^39^ (Spain)	*N* = 172; Mean Age = 48.1, SD = 10.7; 48.3% Female; First episode of psychosis and DSM-5 diagnosis of schizophrenia, schizophreniform, brief psychotic disorder, schizoaffective disorder, bipolar disorder, major depressive disorder, or psychosis NOS at follow-up	L (20 years)	14	Suicidal behavior (attempts)	Number of suicide attempts over 20-year period, dichotomized (none or 1+)	MCCB Social Cognitive Measure—Mayer-Salovey Caruso Emotional Intelligence Test (MSCEIT); Assessed at follow-up	No significant association of social cognition and suicide attempts at follow-up.
Depp et al^40^ (United States)	*N* = 179; Mean Age = 42.1, *SD* = 12.3; 34.6% Female, DSM-IV diagnosis of schizophrenia or schizoaffective disorder	L (2 weeks)	14	SI	Beck Depression Inventory (BDI; Self Report) Item 9—dichotomized presence or absence of SI	Bell Lysaker Emotion Recognition Task	People with SI were more accurate in identifying negative affect (Cohen’s *D* = 0.411) No differences by SI on overall performance or positive affect No relationship of task to SI at 2-week follow-up
						Penn Emotion Recognition 40	No relationship of task to SI at baseline or 2-week follow-up
Dickhoff et al^41^ (The Netherlands)	*N* = 593–715^b^; Mean Age = 27.2, SD = 7.4; 27.5% Female; DSM-IV criteria for a psychotic disorder	L (3 years)	13	SI Suicidal behavior (attempts)	Camberwell Assessment of Need Short Appraisal Schedule (Interviewer-rated)—dichotomized SI Asked participants if they had attempted suicide (interviewer-rated)—dichotomized	Degraded Facial Affect Recognition Task (DFAR); Controlled for general facial recognition ability using the Benton Facial Recognition Task	People who had SI and/or who had attempted suicide had higher scores on the overall DFAR, *F*(1,586) = 4.1, *P* = .04, and for recognizing fearful faces on the DFAR, *P* = .046, η^2^ = 0.007, than those who did not have SI No relationship of the DFAR and future suicide attempts
Harenski et al^42^ (United States)	*N* = 41; Mean Age = 39.7, *SD* = 10.8; 0% Female; DSM-IV diagnosis of a psychotic disorder, criminal offenders	CC	13	Suicidal behavior (attempts)	Columbia Suicide Severity Rating Scale (interviewer-rated)—defined as a “potentially self-injurious act committed with at least some wish to die as a result of the act”	Viewed 13 video clips, selected which emotions the target experienced	Criminal offenders with a psychotic disorder and a history of a suicide attempt had lower emotion recognition scores compared to those without suicide attempts, *P* = .032
Liu et al^43^ (China)	*N* = 301; Mean Age = 33.93, SD = 6.37; 39.2% Female; DSM-5 diagnosis of schizophrenia	CS	12	SI	Beck Scale for SI—Chinese Version; dichotomized to presence or absence of SI	Interpersonal Reactivity Index (IRI)—Affective empathy subscale including empathic concern (other-oriented) and personal distress (self-oriented)	Those with SI had higher IRI personal distress subscale score than people without SI, (22.46 (5.19) vs 20.47 (4.72), *P* = .002). No other significant differences between people with and without SI on IRI subscales
Sastre-Buades et al^44^ (Spain)	*N* = 190; Mean Age = 27.9, *SD* = 7.35; 32.8% Female; DSM-IV criteria for schizophrenia, psychotic disorder NOS, delusional disorder, schizoaffective disorder, brief psychotic disorder, and schizophreniform disorder	CS	14	Suicidal behavior (attempts)	Dichotomized presence of a suicide attempt, defined as “any act of self-harm performed with the intention of taking one’s life”	Faces Test	No significant difference between people who had and had not attempted suicide
Villa et al., 2018^33^ (United States)	*N* = 101; Mean Age = 49.4, *SD* = 11.3; 54.5% Female; DSM-IV criteria for psychotic disorder	CS	13	SI Suicidal behavior (attempts)	Modified Scale of SI (interviewer-rated)—assessed SI within previous 48 h Columbia Suicide Severity Rating Scale (interviewer-rated)—examined lifetime SI and suicide attempts	Penn Emotion Recognition task (ER40)	ER40 total scores and accuracy for specific emotions were unrelated to SI or history of attempt
Wang et al^45^ (China)	*N* = 505^c^; Mean Age = 47.4 *SD* = 12.8; 37% Female; DSM-IV criteria for schizophrenia	CS	14	Suicidal behavior (attempts)	Interviewer asked “Have you ever tried suicide in your lifetime?” Coded as yes or no	Interpersonal Reactivity Index (IRI)—Affective empathy subscale including empathic concern (other-oriented) and personal distress (self-oriented)	People who attempted suicide had a higher “personal distress” subscale score than those who had not attempted suicide, *F*(1,503) = 5.045, *P* = .020 In a regression including IRI total score and subscale scores controlling for age, sex, education, and positive and negative symptoms, only personal distress significantly related to suicide attempt presence, *F*(1,455) = 5.446, *P* = .020
Wastler et al^46^ (United States)	*N* = 65; Mean Age = 23.06; *SD* = 4.34; 29.2% Female; DSM-IV TR Schizophrenia spectrum or affective disorder with psychotic features, first episode psychosis	CS	11	Suicide Risk (Scale included SI and attempt)	Item 8 on the Calgary Depression Scale for Schizophrenia; 0 = Absent; 1 = SI; 2 = SI with Plan; 3 = Suicide Attempt (Unclear if recent or lifetime suicide attempt)	MCCB Social Cognitive Measure—Mayer-Salovey Caruso Emotional Intelligence Test (MSCEIT)	Reported a nonsignificant correlation between suicide risk and the MSCEIT, *r* = 0.116
Yin et al^47^ (China)	*N* = 159; Mean Age = 27.1, *SD* = 8.1; 52.2% Female; DSM-IV criteria for schizophrenia, first episode schizophrenia	CS	14	SI Suicidal behavior (attempts)	Interviewer asked, “Have you ever had thoughts of suicide or worked out a plan in your whole life?” and “Have you ever attempted suicide in your lifetime?”	MCCB Social Cognitive Measure—Mayer-Salovey Caruso Emotional Intelligence Test (MSCEIT)	Compared 3 groups (lifetime SI, lifetime suicide attempt, neither SI nor suicide attempt) and found no group differences on the MSCEIT (controlling for education, depression, and psychiatric symptoms) Robust linear regression showed no significant relationship between suicide and the MSCEIT (controlling for education, depression, and psychiatric symptoms)

*Note.* CC, case control; CS, cross-sectional; DSM-IV, Diagnostic and Statistical Manual IV; L, longitudinal; MCCB, MATRICS Cognitive Consensus Battery; NOS, not otherwise specified.

^a^Indicates that study is a Letter to the Editor.

^b^Sample sizes varied in this study based on the social cognitive task used, characteristics reported only for larger sample.

^c^Full sample is *N* = 627, but only *N* = 505 completed the social cognitive tasks. Characteristics only available for full sample of *N* = 627.

### Method for Assessing Study Quality and Risk of Bias

As the studies in this review include a variety of designs, quality was assessed using a scale developed to assess study quality across varied types.^48,49^ Specifically, this measure addressed whether authors reported information about the aims of the study, if participants declined, inclusion criteria, participant characteristics, ethics approval, power, statistical appropriateness, effect size, and limitations. Scores ranged from 0 to 18, with higher scores reflecting better quality.

## Results

### Included Studies

**Fig. 1.**
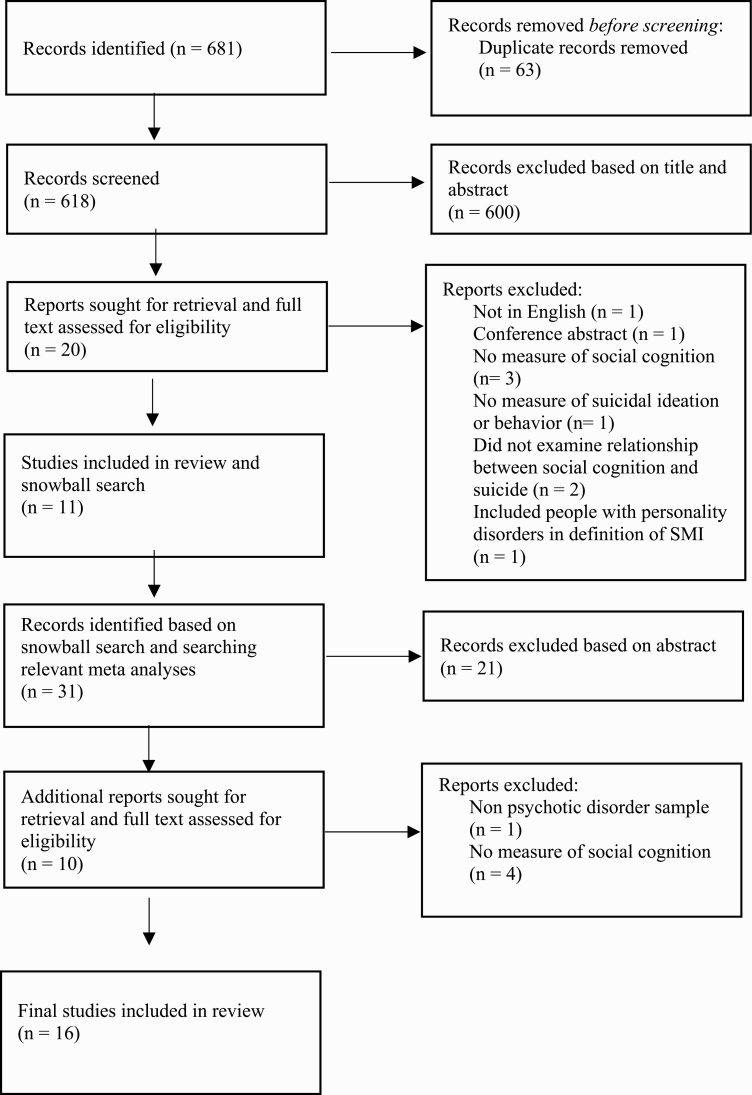
PRISMA flow diagram.

The search process is depicted in figure 1. After removing duplicates, the initial search yielded 618 articles and 600 were excluded after reading the title abstract. This left 20 studies that were screened in phase 2, which after a full text screen left a total of 11 included articles. These articles were searched for additional articles and 2 relevant meta-analyses were examined.^30,31^ Through this process 31 additional records were identified, 5 of which were included, bringing the final number of studies included in this review to 16.

The 16 studies varied in their objectives. The primary objective of 10 of the studies examined the relationship between some aspect of social cognition and SI or behavior,^40–45,50–54^ whereas 5 of the studies did not have a primary objective of examining this relationship and reported statistics examining the relationship.^33,39,46,47,55^ Nine studies included purely cross-sectional analysis of social cognitive variables,^33,43–47,53–55^ 5 studies were longitudinal with follow-up intervals ranging from 2 weeks to 20 years,^39–41,51,52^ and 2 studies were cross-sectional case-control designs.^42,50^ One included study is a Letter to the Editor that contains empirical data not published elsewhere, but is not a peer-reviewed journal article.^53^

### Assessment of Quality

See table 1–3

for full assessment of quality. Study quality ranged from a score of 6 to 15 (mean = 12.4, *SD* = 2.4). Of note, only 1 study reported the number of participants who declined study participation.^47^ Two studies were of relatively low quality, with quality ratings of 6^53^ and 8.^54^ However, due to the exploratory nature of this review, the results of these studies were not excluded. No study was of such a low quality that it was removed from analysis.

### Participant Characteristics

Of the 16 studies included, 2 had overlapping samples.^51,52^ Thus, this review encompasses 15 independent samples and includes a total of *N* = 2631 individuals with a diagnosis of SMI, and sample sizes ranged from *N* = 20 to *N* = 593 (mean = 175.4). Mean ages of the samples ranged from 23.06 to 49.4, and samples varied greatly in their gender distribution, with 1 study only including men^42^ and another study only including women.^50^ Gender distributions of the other included studies were more balanced between men and women. Samples were racially and ethnically diverse, though 8 studies did not report the racial and ethnic makeup of their sample.^39,41,43–45,47,50,54^ Studies came from a diverse range of countries, including the United States (*N* = 5), Spain (*N* = 5), China (*N* = 3), Egypt (*N* = 1), Italy (*N* = 1), and the Netherlands (*N* = 1). Four articles examined a sample of individuals with an FEP,^46,47,51,52^ and 1 study recruited individuals who had experienced an FEP and examined the link between social cognition and suicide 20 years later.^39^

### Assessment of SI and Behavior

Nine studies included a measure of SI.^33,40,41,43,46,47,50,53,55^ Twelve studies collected data relating to SB, specifically suicide attempts.^33,39,41,42,44–47,51,52,54,55^ For SI and behavior, studies varied in the method of assessment (interviewer-rated vs self- or informant-report) and depth (scale vs single item) in characterizing this construct. Importantly, no studies separately analyzed data on types of SB other than actual suicidal attempts (ie, preparatory behaviors, aborted attempts, and interrupted attempts), and only 1 study^55^ examined suicide attempt lethality. One study combined ideation and behavior in their analyses, reporting on characteristics of participants who had made a suicide attempt and/or had SI.^41^ Another study included an interviewer-rated measure of “suicide risk,” which combined SI and a suicide attempt in a single item.^46^

### Measures of Social Cognition

There were 14 social cognitive tasks utilized across the studies, with some studies reporting data from multiple social cognitive tasks, covering domains of emotion recognition/processing, theory of mind, and attributional style or bias. There were no studies that included measures of social perception. The majority of studies utilized well-established social cognitive tasks; however, 1 task was developed for the specific study,^42^ and another study adapted 4 Theory of Mind tasks used for people with Autism.^54^ Of note, some of these tasks (eg, Penn ER40) examine more basic emotion recognition, whereas others (eg, Mayer-Salovey Caruso Emotional Intelligence Test, MSCEIT) examine higher-order emotion processing. Additionally, several papers utilizing the MATRICS Cognitive Consensus Battery (MCCB) did not specifically name the MSCEIT task,^39,45,53^ though the MSCEIT is the measure of social cognition included in the MATRICS battery and was analyzed separately in these papers.^56^

### SI and Social Cognition

#### Emotion Recognition and Processing.

See table 1 for all data. One study that used the Bell-Lysaker Emotion Recognition Task (BLERT) found that participants with SI were more accurate in identifying negative affect, though there were no differences in overall emotion recognition performance.^40^ However, there was no relationship of this task to SI after a 2-week follow-up window.^40^ Additionally, 1 large (*N* = 593) longitudinal study with a 3-year follow-up that used the Degraded Facial Affect Recognition (DFAR) task found that people who had SI and/or a suicide attempt had higher scores on the DFAR, and specifically for recognizing fearful faces, than those who did not have SI.^41^ Two studies used the Penn Emotion Recognition 40 (Penn ER40) and found no relationship of this measure to SI.^33,40^ Therefore, while findings in the literature are mixed, some large and longitudinal studies suggest that SI may be more linked to greater accuracy in recognizing negative affect (eg, fear, sadness) than general emotion recognition ability,^40,41^ though there is no consistent evidence of this link.

Three studies utilized the MSCEIT.^57^ One study found that SI severity was significantly correlated with greater impairment on the MSCEIT, but did not find group-level differences in MSCEIT performance based on SI among participants with schizophrenia.^53^ Two studies including people who had experienced FEP found no differences on MSCEIT performance between people with and without lifetime SI,^47^ and no relation between suicide risk and the MSCEIT.^46^ However, both studies intermixed SB in their measure of SI and/or suicide risk. Additionally, 1 study found that participants with SI had higher affective empathy in the Interpersonal Reactivity Index (IRI) “personal distress” subscale, which measures one’s ability to detect their own emotions.^43^ While the results are mixed, it is possible that emotion regulation and processing is linked to SI severity rather than the presence of SI, as SI severity may be a more sensitive measure than a dichotomous variable.

#### Theory of Mind.

See table 2 for all data. The relationship between theory of mind and SI was examined in 3 studies. In a small sample (*N* = 20) of women using the Reading the Mind in the Eyes Task (RMET), 1 study found that greater scores on the hopelessness subscale of the Suicide Probability Scale was associated with worse performance on the RMET.^50^ However, neither the broader Suicide Probability Scale score nor the SI subscale were correlated with the RMET.^50^ A different longitudinal study in a much larger sample (*N* = 593) using the Hinting Task found that people with SI and/or a suicide attempt had higher scores in theory of mind.^41^ However, in a third study utilizing the IRI cognitive empathy subscales, there was no significant difference between people with and without SI.^43^ Though there is limited literature examining the link between theory of mind and SI in people with psychotic disorders, it appears that people with SI may have higher social cognitive abilities in this domain.

#### Attributional Style and Bias.

See table 3 for all data. Three studies assessed the relationship between attributional bias and social cognition in psychotic disorders. Two studies used the Ambiguous Intention Hostility Questionnaire (AIHQ).^40,55^ While 1 study found that participants with SI had greater attributions of blame in ambiguous situations compared to those without SI,^40^ the other included study found no relationship between the AIHQ blame subscale and lifetime SI.^55^ It is possible that this discrepancy in findings is present as the study led by Depp et al assessed for current, not lifetime, SI. This study also examined the Trustworthiness Task and found no overall performance differences based on SI status and no relationship of the task to SI at 2 weeks, though they found that people with current SI had greater untrustworthiness ratings for the faces in the task that were normatively determined to be untrustworthy. Additionally, 1 study used the DFAR to measure bias and found no relationship between emotion recognition bias, or misidentifying a neutral face as fearful, happy, or angry, and SI and/or a suicide attempt.^41^ The findings in this area suggest that current SI may be more related to attributional style and bias than lifetime SI among people with psychotic disorders.

### SB and Social Cognition

#### Emotion Recognition and Processing.

See table 1 for all data. Five studies examined the relationship between emotion recognition and suicide attempts with mixed results. One large (*N* = 593) longitudinal study with a 3-year follow-up found that people with SMI who had SI and/or attempted suicide had higher scores for recognizing faces in general and fearful faces on the DFAR than those who had not attempted suicide, but no relationship to future suicide attempts.^41^ Similarly, another study found that participants who had attempted suicide had higher affective empathy, especially in the “personal distress” subscale that examines self-oriented ability to detect emotions.^45^ However, 1 smaller study (*N* = 41) including a sample of men who were criminal offenders reported diminished emotion recognition ability is related to suicide attempts in a video emotion identification task.^42^ Despite this, other studies found no relationship between the ER40 and suicide attempts,^33^ or the Faces Test and suicide attempts.^44^ In terms of more complex emotion recognition and processing through the MSCEIT, 1 study found no group differences on MSCEIT performances between people with and without a lifetime suicide attempt in a FEP sample,^47^ and another study found no group differences on MSCEIT performances between people who had and had not attempted suicide over a 20-year follow-up post FEP.^39^ Once again, the literature in this area is mixed, but it is possible that negative affect emotion recognition abilities may be linked to suicide attempts in this population.

#### Theory of Mind.

See table 2 for all data. Five studies examined the relationship between suicide attempts and theory of mind. The False Belief Task was used in 2 studies in an overlapping sample of people who had experienced a FEP,^51,52^ finding that people who attempted suicide made more first-order (inferences about the state of the world) and second-order (inferences about others) false belief errors. In particular, these studies found that first-order false belief errors were related to the presence of a suicide attempt^52^ and the number of suicide attempts over a 1-year period.^51^ In contrast, 1 study examining theory of mind and suicide attempts found a relationship between the likelihood of a lifetime suicide attempt and poorer performance on second-order theory of mind tasks, but not first-order theory of mind.^54^ Two studies utilizing the Hinting Task found no relationship of this task to suicide attempts,^44,52^ though one of these studies demonstrated a small-medium effect size showing that people who had attempted suicide had better performance on this task.^44^ Additionally, another longitudinal study in a larger sample of people with SMI (*N* = 593) found that people who had SI and/or had attempted suicide had higher scores on the Hinting Task than those who had not attempted suicide.^41^ Finally, another large study (*N* = 505) found that people who had attempted suicide had greater degrees of cognitive empathy, particularly the “fantasy” subscale of the IRI (imagining the emotions of actions of book and movie characters), than those who had not attempted suicide.^45^ It is possible that different dimensions of theory of mind are related to suicide attempts in different ways. More errors in inferences about the state of the world and other people may be linked to a greater risk of suicide attempts among people with SMI. Though there is conflicting literature regarding the relationship between theory of mind and lifetime suicide attempts in psychotic disorders, larger studies support that a greater ability to infer the state of mind of others is linked to suicide attempts.

**Table 2.** Studies Reporting on Theory of Mind: Sample Characteristics, Design, Objective, Measurement, and Key Findings

Study and Country of Origin	Sample Size and Characteristics of Group With Psychotic Disorder	Design	Quality Rating	Measured SI and/or Behavior	Measure of SI and/or Behavior	Measure of Theory of Mind	Key Findings—Relationship Between SI and Behavior
Abdo et al^50^ (Egypt)	*N* = 21; Mean Age = 30.2, *SD* = 5.7; 100% Female; DSM-IV criteria for schizophrenia	CC	11	SI/Probability	Suicide Probability Scale (Self Report)—Subscales of hopelessness, SI, negative self-evaluation, and hostility	Reading the Mind in the Eyes Test (RMET)	Hopelessness subscale was associated with worse performance on the RMET (*r* = −0.526, *P* = .017) SI subscale and full suicide probability scale were not statistically significantly correlated with the RMET
Canal-Rivero et al^52^ (Spain)^a^	*N* = 65; Mean Age = 26.2, *SD* = 9.5; 32.3% Female; DSM-IV criteria for affective or non-affective psychosis, first episode psychosis	L (1 year)	13	Suicidal behavior (attempt yes/no; at baseline)	Schedules for Clinical Assessment in Neuropsychiatry (Interviewer-Rated)—Recorded attempts dichotomously	False Belief Task First-Order Inferences about the World; Second-Order Inferences about Others	Higher proportion of people who attempted suicide who made errors on first—(*X*^2^ = 3.95, *df* = 1, *P* = .04) and second—(*X*^2^ = 6.27, *df* = 1, *P* = .04) order false belief tasks First-order false belief errors related to suicide attempts in a regression model controlling for symptom severity and schizoid personality traits (OR = 4.26, 95% CI = 1.05–17.31, *P* = .04)
						Hinting Task	Relationship between the Hinting Task and suicide attempts was not significant
Canal-Rivero et al^51^ (Spain)^a^	*N* = 65; Mean Age = 26.2, *SD* = 9.5; 32.3% Female; DSM-IV criteria for affective or non-affective psychosis, first episode psychosis	L (1 year)	13	Suicidal behavior (number of attempts; 1-year follow-up of Canal-Rivero et al^52^)	Schedules for Clinical Assessment in Neuropsychiatry (Interviewer-Rated)—Recorded attempts dichotomously	False Belief Task First-Order Inferences about the World; Second-Order Inferences about Others	People who made errors in first—(*U* = 310.0, *P* = 0.04) and second—(*U* = 309.5, *P* = .02) order false belief tasks had more suicide attempts than people who did not make errors on the respective task over a 1-year follow-up First-order false belief task errors was significantly related to suicide attempts in a regression model controlling for symptom severity (*B* = 0.49, *t* = 2.11, *P* = .04) over a 1-year follow-up
Duñó et al^54^ (Spain)	*N* = 57; Mean Age = 31.3, *SD* = 8.1; 29.8% Female; DSM-IV criteria for schizophrenia	CS	8	Suicidal behavior (attempts)	Interviews with first-degree relatives and a check of medical records; presence or absence of lifetime suicide attempt	Four false belief Theory of Mind tasks—2 first-order; 2 second-order; Performance dichotomized into Poor and Good	No significant relationship of lifetime suicide attempt to first-order Theory of Mind (OR = 1.62, 95% CI 0.38–6.96). Significant relationship between likelihood of lifetime suicide attempt and poor performance on second order Theory of Mind (OR = 4.02, 95% CI 1.18–13.62) People who made a suicide attempt in their lifetime had poorer performance on second order Theory of Mind (*X*^2^ = 5.223, *P* = .022
Dickhoff et al^41^ (The Netherlands)	*N* = 593–715^b^; Mean Age = 27.2, *SD* = 7.4; 27.5% Female; DSM-IV criteria for a psychotic disorder	L (3 years)	13	SI Suicidal behavior (attempts)	Camberwell Assessment of Need Short Appraisal Schedule (Interviewer-rated)—dichotomized SI Asked participants if they had attempted suicide (interviewer-rated)—dichotomized	Hinting Task	People who had SI and/or who had attempted suicide had higher scores on the hinting task than those who did not have SI, *F*(1,644) = 4.4, *P* = .04 No relationship of the hinting task and future suicide attempts
Liu et al^43^ (China)	*N* = 301; Mean Age = 33.93, *SD* = 6.37; 39.2% Female; DSM-5 diagnosis of schizophrenia	CS	12	SI	Beck Scale for SI—Chinese Version; dichotomized to presence or absence of SI	Interpersonal Reactivity Index (IRI)—Cognitive empathy subscale including perspective taking and “fantasy”	No significant difference between people with and without SI on these subscales of the IRI
Sastre-Buades et al^44^ (Spain)	*N* = 190; Mean Age = 27.9, *SD* = 7.35; 32.8% Female; DSM-IV criteria for schizophrenia, psychotic disorder NOS, delusional disorder, schizoaffective disorder, brief psychotic disorder, and schizophreniform disorder	CS	14	Suicidal behavior (attempts)	Dichotomized presence of a suicide attempt, defined as “any act of self-harm performed with the intention of taking one’s life”	Hinting Task	No significant difference between people who had and had not attempted suicide Small to medium effect size showing that people who had attempted suicide had better performance (*t* = 2.04, *d* = 0.403)
Wang et al^45^ (China)	*N* = 505^c^; Mean Age = 47.4 *SD* = 12.8; 37% Female; DSM-IV criteria for schizophrenia	CS	14	Suicidal behavior (attempts)	Interviewer asked “Have you ever tried suicide in your lifetime?” Coded as yes or no	Interpersonal Reactivity Index (IRI)—Cognitive empathy subscale including perspective taking and “fantasy”	People who attempted suicide had generally higher IRI total (*F*(1,503) = 8.746, *P* = .003) and a higher “fantasy” subscale score (*F*(1,503) = 10.445, *P* = .001) than those who had not attempted suicide

*Note.* CC, case control; CS, cross-sectional; DSM-IV, Diagnostic and Statistical Manual IV; L, longitudinal; NOS, not otherwise specified.

^a^Indicates that samples overlap.

^b^Sample sizes varied in this study based on the social cognitive task used, characteristics reported only for larger sample.

^c^Full sample is *N* = 627, but only *N* = 505 completed the social cognitive tasks. Characteristics only available for full sample of *N* = 627.

#### Attributional Style or Bias.

See table 3 for all data. Three studies examined the relationship between attributional style and SB. One study (*N* = 96) found no relationship between the AIHQ blame subscale, history of a lifetime suicide attempt, or suicide attempt lethality.^55^ Another study used the DFAR and found no relationship between emotion recognition bias and SI and/or a suicide attempt.^41^ A third study utilized the Internal Personal and Situational Attributions Questionnaire and found no significant difference between people who had and had not attempted suicide on this measure, though they demonstrated a small-medium effect size showing that people who had not attempted suicide had a greater externalizing bias than those who had attempted suicide.^44^ While attributional style or bias may be linked to SI, it does not appear to be linked to SB in psychotic disorders, but this finding should be interpreted with caution due to a limited number of available studies.

**Table 3.** Studies Reporting on Attributional Style and Bias: Sample Characteristics, Design, Objective, Measurement, and Key Findings

Study and Country of Origin	Sample Size and Characteristics of Group With Psychotic Disorder	Design	Quality Rating	Measured SI and/or Behavior	Measure of SI and/or Behavior	Measure of Attributional Style or Bias	Key Findings—Relationship Between SI and Behavior
Chalker et al^55^ (United States)	*N* = 96; Mean Age = 43.9, *SD* = 11.2; 55.2% Female; DSM-5 criteria for affective or non-affective psychosis	CS	15	SI Suicidal behavior (attempts)	Columbia Suicide Severity Rating Scale (Interview-Rated)—Recorded lifetime and current SI, dichotomous attempts, number of attempts, and suicide attempt lethality	Ambiguous Intentions Hostility Questionnaire (AIHQ) Blame Subscale	No relationship found between the AIHQ blame subscale and history of any lifetime SI, history of active SI with a plan or intent, history of lifetime suicide attempt, and suicide attempt lethality
Depp et al^40^ (United States)	*N* = 179; Mean Age = 42.1, *SD* = 12.3; 34.6% Female, DSM-IV diagnosis of schizophrenia or schizoaffective disorder	L (2 weeks)	14	SI	Beck Depression Inventory (BDI; Self Report) Item 9—dichotomized presence or absence of SI	Ambiguous intentions hostility questionnaire (AIHQ)—Blame, Hostility and Aggression Subscales	Participants with SI had a higher blame score, when controlling for general and positive symptoms (Cohen’s *D* = 0.433) Only the blame subscale (vs hostility and aggression subscales) was related to SI (Cohen’s *D* = 0.599) Baseline AIHQ related to SI at 2-week follow-up (OR = 1.20, CI = 1.05–1.39, *P* = .010)
						Trustworthiness Task	No overall task performance differences by SI status People with SI had greater untrustworthiness ratings for the faces deemed to be most normatively untrustworthy (Cohen’s *D* = 0.390) No relationship of task to 2-week follow-up
Dickhoff et al^41^ (The Netherlands)	*N* = 593–715^a^; Mean Age = 27.2, *SD* = 7.4; 27.5% Female; DSM-IV criteria for a psychotic disorder	L (3 years)	13	SI Suicidal behavior (attempts)	Camberwell Assessment of Need Short Appraisal Schedule (Interviewer-rated)—dichotomized SI Asked participants if they had attempted suicide (interviewer-rated)—dichotomized	Degraded Facial Affect Recognition Task (DFAR); Bias Measure—seeing a Happy, Fearful, or Angry Face when a Neutral face was shown	No difference between people who had SI and/or who had attempted suicide on misperception of emotional faces
Sastre-Buades et al^44^ (Spain)	*N* = 190; Mean Age = 27.9, *SD* = 7.35; 32.8% Female; DSM-IV criteria for schizophrenia, psychotic disorder NOS, delusional disorder, schizoaffective disorder, brief psychotic disorder, and schizophreniform disorder	CS	14	Suicidal behavior (attempts)	Dichotomized presence of a suicide attempt, defined as “any act of self-harm performed with the intention of taking one’s life”	Internal Personal and Situational Attributions Questionnaire—includes personalizing and externalizing subscales	No significant difference between people who had and had not attempted suicide Small to medium effect size showing that people who had not attempted suicide showed more externalizing bias than those who had attempted suicide (*t* = 2.07, *d* = 0.482)

*Note.* CS, cross-sectional; DSM-IV, Diagnostic and Statistical Manual IV; L, longitudinal; NOS, not otherwise specified.

^a^Sample sizes varied in this study based on the social cognitive task used, characteristics reported only for larger sample.

## Discussion

This systematic review indicates heterogeneous effects of social cognitive domains as they relate to SI and SB, with variation in the direction of effects as well as across social cognitive domains. The studies included a diverse range of individuals with current or lifetime SI and/or past suicide attempts. While the findings were mixed, larger and longitudinal studies suggest that SI and attempts are linked to greater accuracy in recognizing negative affect (eg, fear, sadness). While the data is limited, the theory of mind data suggests that people with SMI and SI have higher abilities in this domain; this was also true for those with a lifetime suicide attempt. Additionally, current vs lifetime SI was more strongly related to attributional style and bias and may not be related to suicide attempts or lethality of suicide attempts. This review also highlighted several inconsistencies in this literature that make it difficult to draw conclusions, such as sample variability, inconsistent assessment tools, and the limited number of longitudinal studies.

Related to SI, though the literature is mixed, it appears that emotion recognition of negative affect is linked to the presence of SI,^40,41^ potentially more so than emotion recognition abilities as a whole.^33,40^ Relatedly, it appears that attributional bias may be linked to current SI,^40^ but not lifetime SI.^41,55^ Overall, it seems that SI may be linked to a bias toward negative appraisals of emotion, whether it be through a bias toward recognizing negative affect and discounting positive affect, or through attributional styles attributing negative intentions to others. It is possible there is a predilection to negative affect recognition given the link between negative affect and SI, particularly documented in children and adolescents.^58–60^ This may be particularly relevant for those experiencing psychosis given the likely onset of psychotic-like experiences in adolescence and early adulthood.

Contrary to the results of a recent meta-analysis showing that theory of mind deficits were linked to greater SI and SB in a broad clinical population,^30^ the results of this review suggest that in people with SMI, better theory of mind abilities may be linked to SI.^41^ This is consistent with studies showing that better neurocognitive ability is associated with SI and behavior among people with psychotic disorders, but in contrast with other clinical populations in which the association between neurocognitive ability and SI is negative.^61–64^ Some have speculated that better neurocognitive cognitive ability may be linked to higher self-reflectiveness, which may be related to greater SI, however, the relationship between cognition and self-reflectiveness has not been established.^62^ It is unclear whether this hypothesis may extend to the relationship between social cognition and SI, which is a question for future study. Future studies in this area should aim to further clarify the specifics of this counter-intuitive relationship, including greater specificity in the domains and social cognitive skills that may be linked to greater SI and SB in this population, the relationship of social cognitive ability to insight and self-reflectiveness, as well as identifying other clinical and psychological correlates to this relationship.

Similar to SI, the literature in relation to SB is mixed, but it is possible that domains of emotion recognition, specifically negative affect, are linked to suicide attempts^41^ more than general emotion recognition abilities.^33,44^ Regarding theory of mind, it appears that more errors in inferences about the state of the world are linked to suicide attempts,^51,52^ whereas similar to SI, a greater ability to infer the states of mind of others is linked to suicide attempts.^41,45^ This review provided little evidence for a relationship between attributional biases and SB, however, there were only 3 studies that examined this relationship. Additionally, 2 studies found a link between suicide-related variables and affective empathy, specifically that people with SI^43^ or who had attempted suicide^45^ had higher scores on a measure of personal distress, or the ability to recognize one’s own emotions. Overall, though this literature is mixed, the relationship between social cognition and SB mirrors the relationship to SI, with a particular focus on the link of greater ability to identify negative affect and greater theory of mind abilities and increased SB.

The limitations in this review include exclusion of people without a diagnosis of SMI (eg, people at clinical high risk for psychosis, people with psychotic-like experiences). While this method was chosen to focus on people with full-threshold psychotic symptoms, it limits how conclusions can be applied to those with subthreshold psychotic symptoms. Additionally, we were unable to include or access unpublished data in this review, which may be useful to clarify the present mixed findings. The included studies are limited in their measurement of SI and SB. For instance, 2 studies only used 1 item to examine SI,^40,45^ 2 studies included non-suicidal self-injury in with SB,^51,52^ and 1 study included aborted and interrupted attempts with SI.^47^ One study combined measures of SI and behavior, limiting the conclusions that could be drawn about each construct separately.^41^ Furthermore, no studies examined other forms of SB such as preparatory behaviors. Future research should incorporate a dimensional view of SI in order to sensitively capture a spectrum of the construct, doing so using well-validated measures such as the Modified Scale of SI (MSSI)^65^ or the Columbia Suicide Severity Rating Scale (CSSRS).^66^ Additionally, future studies should consider nuances of other SBs through these validated measures.

Some studies are also limited in their measurement of social cognition, as no studies utilized a measure of social perception, and 1 study used a task that has not been validated in people with SMI.^42^ Furthermore, the tasks of social cognition varied across studies, compromising the ability to generalize across studies. Many studies utilized social cognitive measures that were not evaluated by the Social Cognition Psychometric Evaluation study (SCOPE),^21,67^ which systematically evaluated the psychometric properties of social cognitive measures in a large sample of people with schizophrenia. This calls into question the reliability and validity of the measures utilized, and limitations in the measurement of social cognition may partially explain the mixed findings in the present review. Thus, future studies should consider examining the link between social perception and SI and SB in populations with SMI and should also aim to use well-validated measures recommended by large studies of social cognition in schizophrenia, such as the SCOPE study.^20,21^ The inclusion of more reliable and valid measures of social cognition will allow for a more nuanced understanding of the specific domains and skills related to SI and SB in this population. Additionally, within domains, it is unclear how other psychological processes such as hypermentalization (ie, over-attributing the mental states of others)^68^ may relate to social cognition or IPTS constructs such as perceived burdensomeness or thwarted belongingness. The measurement of this construct and its association with social cognition and IPTS constructs in this population is a direction for future study.

Additionally, only 5 studies included longitudinal data,^39–41,51,52^ and only 3 of these studies examined the relationship between baseline social cognition and later SI and/or behavior with follow-up intervals of 2 weeks,^40^ 1 year,^51^ and 3 years.^41^ If possible, longitudinal studies should understand the relationship of social cognition to future suicide attempts prospectively to further elucidate the relationship between social cognition and later development of SI or SB (eg, see CSR&D VA Merit Award CX002485, PI^69^).

Adjacent to the aims of this review, some articles included in the review highlighted other relevant factors for future studies to consider as potential mediators, moderators, or covariates. First, future studies should consider the role of childhood trauma in the relationship of social cognition, SI, and SB. This potential relationship is of particular importance given the incredibly high rate (90%) at which individuals at clinical high risk for psychosis report traumatic events and childhood victimization.^70^ It is well established that childhood trauma may have detrimental effects on psychosocial development, mental health, and feelings of loneliness in adulthood.^71–74^ Furthermore, there is literature suggesting the link between social cognition deficits and higher reports of childhood trauma in schizophrenia compared to healthy adults.^75^ Given the high prevalence of suicidal thoughts and behaviors in psychosis,^76^ it is possible that childhood trauma may impact social cognition which in turn may impact factors related to SI and SB, or vice versa. In the present review, 1 study found that childhood trauma was independently related to SI, SB, and attributional bias.^55^ This suggests that social cognitive biases and/or SI and behavior may stem from childhood trauma in a developmental pathway, but this link has yet to be examined in the literature. Future research should examine the links between childhood trauma, social cognition, and SI and SB, and may aim to understand the directionality of these previously established associations.

Second, 2 studies utilized the Interpersonal Needs Questionnaire (INQ), a common measure of perceived burdensomeness and thwarted belongingness, both major components of the Interpersonal Theory of Suicide (IPTS).^77^ The IPTS posits that perceived burdensomeness, or the idea that you are a burden to others, and thwarted belongingness, or the idea that you do not belong with others, converge with hopelessness and manifest in SI.^77,78^ There is evidence that perceived burdensomeness and thwarted belongingness are linked to emotion recognition deficits among people with psychotic disorders,^33^ and it is possible that social cognitive impairments may be linked to suicide via these interpersonal constructs. The studies from the current review found that the INQ was related to emotion recognition biases^33^ and childhood trauma,^55^ suggesting that perceived burdensomeness and thwarted belongingness relate to childhood trauma and social cognition. It is possible that childhood trauma among people with psychotic disorders may be significantly related upstream of perceived burdensomeness, thwarted belongingness, and social cognitive biases, but this has yet to be examined.

Third, most papers in this review (*n* = 11) included a measure of positive symptoms, most commonly the positive and negative syndromes scale (PANSS, Kay et al^79^). Many studies included positive symptoms as a covariate in models due to its association with SI and SB. Positive symptoms are related to social cognitive deficits as well, as previous literature shows that people with higher levels of paranoia tend to misperceive more neutral faces as angry.^18^ Therefore, future studies should consider interacting dynamics of trauma, perceived burdensomeness, thwarted belongingness, and positive symptoms in the relationship of social cognition and behavior.

Fourth, several studies included in this review (*n* = 9) included some measure of general cognition, yet only 2 papers adjusted analyses of social cognition and SB in some analyses.^39,54^ Future studies should consider controlling for the role of general cognitive ability to understand social cognitive effects over and above generalized cognitive issues.

The findings from this literature review were mixed, but it appears that among people with psychotic disorders, better emotion recognition ability for negative affect, better theory of mind ability, and attributional biases are linked in a nuanced manner to SI and behavior. Despite the potential clinical implications of the extant literature included in this review, additional research is needed before this evidence can be translated to suicide prevention efforts that target social cognitive abilities and biases in SMI. We consider these findings to be preliminary and there is much work to be done to understand the pathways by which these constructs relate to SI and behavior, and what constructs may mediate or moderate these relationships.

## Supplementary Material

sgae007_suppl_Supplementary_Materials
