# Supplementary material for: Systematic Review of the Link Between Social Cognition and Suicidal Ideation and Behavior in People With Serious Mental Illness
Source: Schizophr Bull Open. 2024 Mar 27;5(1):sgae007. doi: 10.1093/schizbullopen/sgae007 (PMC11014866; doi:10.1093/schizbullopen/sgae007)
Supplement: sgae007_suppl_Supplementary_Materials [file sgae007_suppl_Supplementary_Materials.docx]

**Supplementary Material**

Combinations of the following search terms were used for all databases: (psychotic disorders OR psychosis OR schizophrenia OR schizoaffective OR bipolar disorder OR serious mental illness) AND (social cogniti* OR emotion recognition OR emotion perception OR social perception OR theory of mind OR mental state attribution OR attributional style OR attributional bias) AND (suicide OR SI OR suicidal behavior OR suicidality OR suicide attempt OR completed suicide).
